# High-throughput methods for identification of protein-protein interactions involving short linear motifs

**DOI:** 10.1186/s12964-015-0116-8

**Published:** 2015-08-22

**Authors:** Cecilia Blikstad, Ylva Ivarsson

**Affiliations:** Department of Chemistry – BMC, Husargatan 3, 751 23 Uppsala, Sweden

**Keywords:** Linear motif, Protein-protein interactions, Phage display, Yeast-surface display, Peptide arrays, Interaction profiling

## Abstract

Interactions between modular domains and short linear motifs (3–10 amino acids peptide stretches) are crucial for cell signaling. The motifs typically reside in the disordered regions of the proteome and the interactions are often transient, allowing for rapid changes in response to changing stimuli. The properties that make domain-motif interactions suitable for cell signaling also make them difficult to capture experimentally and they are therefore largely underrepresented in the known protein-protein interaction networks. Most of the knowledge on domain-motif interactions is derived from low-throughput studies, although there exist dedicated high-throughput methods for the identification of domain-motif interactions. The methods include arrays of peptides or proteins, display of peptides on phage or yeast, and yeast-two-hybrid experiments. We here provide a survey of scalable methods for domain-motif interaction profiling. These methods have frequently been applied to a limited number of ubiquitous domain families. It is now time to apply them to a broader set of peptide binding proteins, to provide a comprehensive picture of the linear motifs in the human proteome and to link them to their potential binding partners. Despite the plethora of methods, it is still a challenge for most approaches to identify interactions that rely on post-translational modification or context dependent or conditional interactions, suggesting directions for further method development.

## Introduction

The size of the human interactome has been estimated to 650,000 interactions [1]. The known interactome is rapidly growing through the efforts of various high-through put studies such as affinity-purification coupled to mass spectrometry (AP-MS) [2] and yeast-two-hybrid (Y2H) [3]. However, less than 20 % of potential pairwise human protein-protein interactions have been explored through high-throughput studies [4]. About 15–40 % of the protein-protein interactions involve the recognition of a peptide motif (3–10 amino acid stretches) by a globular protein [5]. These interactions have crucial roles in defining cellular functions, being involved in processes such as protein scaffolding, cell signaling, targeting to subcellular compartments and post-translational modifications (PTMs) [6]. In parity with the large number of proposed interactions, a recent estimate suggested that the human proteome holds over 100,000 binding motifs [7]. The motifs are typically found in disordered regions or in exposed flexible loops and bind their target proteins through transient interactions with affinities in the low- to mid-micromolar range [8, 9]. A recent analysis revealed that 22 % of human disease mutations occur in the unstructured regions, and suggested that disease mutations in motifs are neglected players in cancer [10]. It is thus of crucial importance to systematically identify linear motifs in the proteome and link the motifs to the domains that recognize them.

A growing number of domains have been found to engage in peptide-mediated interactions. Today, there are about 200 known peptide binding domain families [11] with well studied examples being the PDZ (postsynaptic density protein 95/discs large/zona occludens 1) domains that typically bind to C-terminal peptides of target proteins [12–14], the poly proline binding WW domains [15] and SH3 (Src Homology 3) domains [16, 17], and the phosphotyrosine binding SH2 (Src Homology 2) domains [18–22] (Table 1). Manually curated databases such as the eukaryotic linear motif (ELM) resource [23] and the Linear Motif mediated Protein interaction Database (LMPID) [24] contain over 2,000 annotated instances of domain-motif interactions, most of which have been discovered by low-throughput experiments such as pulldowns, co-immunoprecipitation (co-IPs), mutational analysis and detailed structural studies of domain-peptide complexes. There is thus a striking discrepancy between the estimated number of motif-based interactions and the experimentally validated cases, suggesting that a vast number of motifs and binding domains are to be discovered. However, domain-motif interactions are difficult to capture due to their limited binding interfaces [8]. They have therefore commonly been overlooked in the methods such high-throughput AP-MS or Y2H. Indeed, an analysis of Y2H data revealed that only 1 % of the interactions rely on interactions with linear motifs [5]. The interactions can however be captured through AP-MS by the use of cross-linking [25] or by a recently developed proximity biotinylation approach [26, 27]. Although these methods may capture transient interactions, they will not necessarily report on binary interactions and they provide no direct information on the motifs that are involved in the interactions.

**Table 1:** Examples of interactions between modular domains and linear motifs

Protein	Domain	Consensus motif	Target protein/binding peptide	Function	Reference
GRB2	SH2	pY-x-(E/N)	ERBB3/pYMN	Ras signaling	[93]
GRB2	SH3	P-x-x-P-x-(R/K)	SOS1/PPVPPR	Ras signaling	[94]
SUMO1	SUMO	(V/I/L)-(V/I/L)-(D/E)-(V/I/L)	PIAS1/VIDL	Sumoylation	[66]
SDCBP	PDZ	Φ-x-Φ-coo-	SDC1/EFYA-coo-	Trafficking	[95]
YAP1	WW	PP-x-Y	TP73/PPPY	Transcriptional regulation	[96]

“Φ” indicates a hydrophic residue and “x” any amino acid

**Fig. 1:**
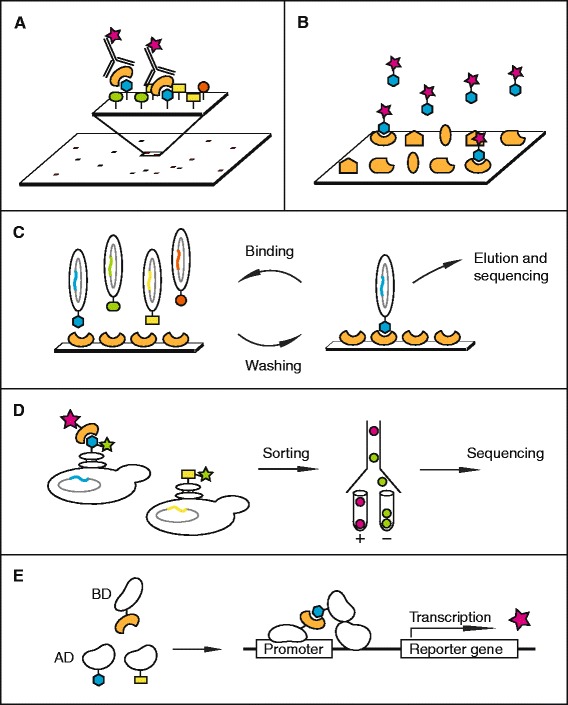
Schematic representation of discussed techniques for the identification of motif-based interactions. Orange represents target protein; blue hexagon represents a binding motif; yellow, green and purple represent non-binding sequences peptides. Pink star represent a detection signal e.g. fluorescence. **a** Peptide microarray: Peptides with known sequences are synthesized on a solid support, incubated with the target protein and interactions are detected with specific antibodies or labeled target protein. **b** Protein array: A selection of different purified proteins are spotted on a solid support and incubated with a labeled peptide. **c** Peptide phage-display: Bait protein is immobilized and used in selections against a peptide phage library. Unbound phage particles are washed away, bound phage eluted and amplified, and used for repeated rounds of selections. Enriched binding clones are sequenced. **d** Yeast surface display: A library of peptides are displayed on the surface of yeast cells and incubated with a target protein. The target protein is labeled with a fluorescent tag and the cells are sorted based on peptide binding using FACS. Sorted pools are sequenced. **e** Yeast-two-hybrid: The binding domain (BD) of a transcription factor is linked to the target protein and the activation domain (AD) of the same transcription factor is linked to a peptide. If the protein and peptide interact BD and AD are brought together and the transcription factor reconstituted. This activates the transcription of a reporter gene

There is a variety of experimental methods dedicated to the characterization of peptide binding modules and the identification of peptide binding motifs [28]. The methods essentially fall into three main categories: arrays, display methods and protein-fragment complementation assays. Here, we summarize these methods for the identification of motif-based interactions (Fig. 1, Table 2); we introduce the basic principle of the methods and highlight recent advances in high-throughput analysis of domain-motif interactions.

**Table 2:** Overview of discussed methods for identification and characterization of motif based interactions

Method	Peptide library size	Pros	Cons
Combinatorial phage display	10^10^	Library size	No PTMs, various biases
ProP-PD	10^4^–-10^6^	Unbiased	No PTMs
Yeast surface display	10^8^	Some PTMs, unbiased	Limited library size
Peptide array	10^2^ – 10^3^	PTMs, semi-quantitative	Limited coverage
			Biased library
			Cost of materials
High-density array	10^5^ – 10^6^	Coverage	Cost of materials
Protein microarray	10–100 s	Semi-quantitative	Protein stability
			Labor intense set-up
Y2H	10^6^	Low tech	No PTMs

### Microarrays

#### Peptide arrays

Peptide arrays rely on the chemically synthesis of peptides with known sequences on a solid support such as a cellulose membrane or a glass slide [29–32]. The microarray is thereafter incubated with the target protein and the bound protein is detected using for example specific antibodies or fluorescent or radioactive labeled proteins (Fig. 1a). Peptide arrays are typically semi-quantitative and allow the comparison of affinities between ligands immobilized on the same slide. An advantage of peptide array over display methods is that the peptide sequences are known and that the sequences can be systematically varied to map binding motifs. The method also provides information on non-binding peptides. A drawback of the method is a high number of false positive and false negative read-outs. This is partly due to the fact that the yield and purity of the peptides are difficult to evaluate and can vary between peptides on the same chip.

Peptide arrays were first introduced in the early nineteen’s when two groups reported techniques for parallel chemical synthesis of peptides on solid support. Fodor and co-workers described a light-directed, spatially addressable parallel chemical synthesis [33] and Frank introduced the SPOT-synthesis [34]. The majority of peptide arrays reported to date have relied on the SPOT-synthesis, which is commercially available and can be performed fully automated. Peptides are typically synthesized with a free N-terminal sequence. However, SPOT arrays have been further adapted for the synthesis of peptides with free C-terminal sequences, which was crucial for probing the binding specificities of, for example, PDZ domains [35].

A main advantage of peptide arrays is the possibility to incorporate modified and non-natural amino acids. This allow for direct and controlled mapping of interactions regulated by PTMs, such as phosphorylation [21] and acetylation [36]. For example, the tyrosine phosphopeptide binding of SH2 domains have been elucidated using a quantitative peptide microarray based approach [18] and by the use of a high-density peptide chip technology [21]. Similarly, Filippakopolous and co-workers created SPOT arrays that covered all possible sites for ε-N-acetylation of lysine residues of human histones [36]. These arrays were screened against 43 members of the bromodomain family. Affinities were determined by isothermal titration calorimetry (ITC) and a comprehensive structural characterization was performed. The study suggested that bromodomains recognize a combination of PTMs rather than single acetylates sequences.

Traditionally the throughput of peptide microarrays has been up to a few thousand peptides per chip. Ultra-dense peptide arrays now allow array sizes of 10^5^–10^6^ peptides [37–39]. These ultra-dense peptide arrays have been used for epitope mapping of antibodies. For example, Uhlen and co-workers developed a proteome wide peptide array, which was used for epitope mapping and cross-reactivity analysis of antibodies [38]. Using a photolithic technique they were able to *in situ* synthesize a total of 2.1 million overlapping peptides. This approach should be applicable for the general purpose of identifying motif-based interactions.

Apart from characterizing binding specificities of purified proteins, peptide microarrays can be used to identify targets from cell lysate. Taking such a motif centric approach, Okada and co-workers identified domains binding to proline rich peptides by synthesizing a peptide array, exposing it to cell lysate, cross-linking and identification of binding proteins through mass spectrometry. Thus, given a set of motifs, it is possible to identify proteins recognizing the given sequences [40].

Taken together, peptide arrays are useful tools for the identification and characterization of motifs-based interactions and are suitable for addressing interactions that rely on PTMs.

### Protein arrays

In protein microarrays (Fig. 1b), proteins of interest are immobilized on a surface and then probed for binding to a labeled protein or peptide [41]. Proteins can be prepared by over-expression and high-throughput purification followed by spotting on the surface, or be obtained by cell-free protein expression systems [42, 43]. Proteomic microarrays allow the investigation of protein-protein interactions on a global scale [44, 45]. Protein microarrays have for example been used to elucidate the peptide binding specificities of the WW domain family [15]. Potential WW binding sites in the human proteome were identified by scanning the proteome using previously known motifs. Representative peptides were synthesized and their binding towards the WW domains tested through a quantitative ELISA-like binding assay. In another study, protein microarrays of SH2 domains and phosphotyrosine-binding (PTB) domains were used to explore their phosphorylation dependent interactions with 61 peptides representing tyrosine phosphorylation sites on the ErbB receptors [20]. Additionally, the specificities of PDZ domains were analyzed through protein microarrays paired with quantitative fluorescence polarization [13]. Protein arrays are thus useful tools for the comparative analysis of binding specificities of peptide binding modules. Among the advantages are the low sample consumption and the possibility to study interactions relying on PTMs. The method can further be used to obtain quantitative information on binding affinities. Among the disadvantages are the labor intense set-up and the requirement for rather high affinity interactions (K_D_ <50 μM) [46].

### Display methods

#### Peptide phage display

Peptide phage display is a powerful tool for the analysis of binding specificities of peptide binding domains [47]. Phages are viruses that infect bacteria. A link between the genotype and phenotype of the phage is provided by inserting DNA inside of the phage that encode for peptides which are displayed on the phage surface. Binding clones are enriched through selections against immobilized bait proteins and are then subjected to sequence analysis (Fig. 1c). There are various phage display systems, with the most commonly used being the p3 or p8 protein of the filamentous M13 phage or the minor coat protein 10B of the lytic T7 phage, as reviewed elsewhere [47]. The display can be either monovalent or multivalent, the former being preferred for capturing stronger interactions and the latter more suited for the identification of weaker interactions due to the avidity of the displayed peptides. The main strength of the method is that it allows the construction of highly diverse peptide libraries (10^10^) at a rather low cost. In a typical combinatorial peptide phage display experiment, libraries display randomized peptide sequences. The bottleneck has traditionally been the sequencing of binding clones. Today, next-generation sequencing brings down the cost of sequencing and the labor, which has opened new possibilities to exploit the potential of phage display and to gain control over the phage library compositions [48].

Peptide phage display has been used to characterize the binding specificities of various domain families. For example, the binding specificities of the yeast SH3 domains were elucidated in 2002, and the results were paired with computational predictions and with a Y2H derived protein-protein interaction network [17]. More than 10 years later, Xin et al. profiled the binding preferences of 36 SH3 domains of *Caenorhabditis elegans* [16], which revealed that the binding preferences were largely conserved between yeast and worm. Also the PDZ domains have been profiled through phage display. Tonikian et al. performed a large-scale characterization of PDZ binding specificities for 54 human and 28 worm PDZ domains [14], which allowed for an extended classification of their binding specificities. This information was later used to identify subspecificities among PDZ domains [49] and was paired with peptide array data [13] to construct a human PDZ domain-ligand interaction network [50].

Combinatorial phage display selections are useful for the identification of high affinity binders and the generation of consensus motifs. However, the displayed peptides may have little to do with biologically relevant targets. A study by Luck et al. highlighted that several of the consensus motifs for PDZ domains derived from combinatorial phage display are overly hydrophobic (i.e. tryptophan rich), which compromises the predictions [51]. Different attempts have been made to create phage libraries that display peptides representing parts of the human proteome, among them cDNA display and open reading frame display [47, 52]. These experiments have typically suffered from low library quality. A recent addition is the proteomic peptide phage display (ProP-PD) where phage libraries are designed to display regions of a target proteome [53, 54]. This method combines microarray synthesis of highly defined oligonucleotide libraries and next-generation sequencing. In 2011, Larman and co-workers created a T7 phage library that displays 36-mer peptides covering the human proteome [54]. More recently, this was followed by a study where M13 phage libraries were created to display the C-terminal peptides of human or viral proteins [53]. The C-terminal ProP-PD libraries were validated against a set of PDZ domains and it efficiently identified binders of potential biological relevance. ProP-PD directly identifies the binding motifs and the host proteins, thus obviating the need for predictions.

Phage display is an efficient approach for the determination of peptide binding specificities, which in case of ProP-PD provides direct information on binding sites in target proteins. Among the main benefits is the possibility to create highly diverse phage libraries and the fact that once a library has been created, it can be used over and over again. The method is suited for unbiased discovery of binding motifs, as no information is required beforehand for designing the phage display libraries. Phage display can be performed in high throughput. In such experiments, protein expression, purification and phage selections are performed in 96-well plates and the retained phage pools are analyzed by next-generation sequencing [55]. The limiting factors for these experiments are the availability of expression constructs, data analysis and the downstream validations. The main limitation of the technique is that it is not suited for capturing interactions that rely on PTMs.

### Yeast surface display

Yeast surface display was developed nearly 20 years ago as a tool for in vitro evolution of proteins [56]. However, the technique can also be used for identification of protein-protein interactions and epitope mapping of antibodies. Similar to phage display, there is a direct link between the genotype and the phenotype [57–60]. Each yeast cell carries plasmid DNA that codes for a peptide that is displayed on the yeast cell surface. Typically, the *Saccharomyces cerevisiae*–Aga2p system is used, where peptides are displayed as fusions with the Aga2p subunit of the mating protein a-agglutinin (Fig. 1d). Aga2p is linked to the Aga1p subunit, via two disulfide bonds, which is anchored to the cell surface. Up to 50,000 copies of the peptide are displayed on a single cell. The cells are incubated with labeled protein and sorted based on binding to the protein using fluorescence-activated cell sorting (FACS) or magnetic-activated cell sorting (MACS). The sorted pools are thereafter sequenced. Signal intensities resulting from binding can be normalized against expression levels of the displayed peptide by concurrently tagging the peptide with a fluorescent tag.

Similar to phage display, next-generation sequencing has opened new possibilities to obtain comprehensive information on binding clones. The combination was for example used to identify unique major histocompability complex peptides that are recognized by T cell receptors [61]. It has also been used to identify peptides that bind to either Mcl-1 or Bcl-xL selectively, or to both with high affinity, by screening a library of randomized BH3 peptides [62]. An advantage of yeast surface display is the possibility to obtain information on non-binding clones. Another significant advantage is that yeast is eukaryotic and the system has some levels of PTMs. The main limitation with yeast surface display is the throughput, which is 100–1000 magnitudes lower than that of phage display.

### Y2H

Y2H was first reported in 1989 [63]. It relies on the splitting of a DNA binding domain and an activation domain of a transcription factor that are linked to a prey or a bait protein. If the bait and prey proteins interact, the two domains of the transcription factor are brought together and the reconstituted transcription factor activate the transcription of reporter genes (Fig. 1e). The assay can be carried out against one prey at a time, or against libraries of prey proteins/peptides. Y2H is currently providing a massive amount of data on protein-protein interactions through the systematic efforts of Vidal and co-workers [3]. The method is in theory capable of capturing interactions relying on motif based interactions, but is in practice largely failing to identify these kinds of interactions [64]. Furthermore, Y2H does typically not provide information on the motifs involved in the identified binary interactions. For example, a large scale Y2H analysis of PDZ domains suggested that many PDZ domains do not rely on a free C-terminal region for binding, however the study did not identify the internal binding motifs [65]. Despite these issues, there are several successful cases of motif profiling through Y2H, such as the successful identification of SUMO interacting motifs for SUMO1 and SUMO2 [66]. In case of PDZ domains, Belotti and co-workers constructed an array for Y2H screening that contain 96 % of the human PDZ domains, and validated it against a select set of C-terminal preys, such as the E6 oncoviral protein and a set of protein kinases [67]. The interactions were further confirmed through mass spectrometry.

Y2H can also be used for characterization of peptide binding motifs by screening random peptide libraries [68]. For example, specificities of five PDZ domains were analyzed by screening of a candidate ligand library using a Y2H mating array [69]. Furthermore, the PDZ proteins PDZK1 and LNX were analyzed through Y2H screening against random peptide libraries [70, 71]. Similarly, the binding preferences for internal PDZ binding motifs was profiled by screening of 24 PDZ domains against a nearly random octapeptide Y2H library [72]. Thus, Y2H can be adopted for domain-motif interaction screening. The main issues with the method are a high percentage of false positives and false negatives read-outs. A particular issue is that the assay requires that proteins can be translocated to the nucleus. Although not reviewed here, there are other split-protein systems that may identify motif-based interactions [73, 74].

### Validations of domain-motif interactions

With the development of high-throughput methods for identification of domain-motif interactions there is a need for high-throughput methods for affinity determination. In addition, if the aim is to identify biologically relevant domain-motif interactions, cell based validations are crucial. Both of these downstream validations may create bottlenecks. Typical methods for affinity determinations such as surface plasmon resonance and ITC provide high quality information, but have limited throughputs. To tackle the issue, various studies have reported methods for high-throughput measurements of protein-peptide interactions. A protocol for high-throughput affinity determinations using a protein microarray and fluorescently labeled synthetic peptides was published by Kaushansky et al. [46]. Moreover, a large-scale fluorescence polarization (FP) methodology using synthetic phosphopeptides was reported for affinity determinations of interactions involving the ErbB receptor phosphosites [19] and Reich et al. described SORTCERY, which is a method for ranking hundreds of yeast-displayed peptides according to their affinities for a target interaction partner [75]. The procedure involves fluorescence-activated cell sorting of a library, next-generation sequencing of sorted pools and computational analysis.

A recent addition is the high-throughput holdup assay [76]. The method is developed for affinity determinations of domain-motif interactions and can measure up to 1,000 binding affinities per day. Essentially, extracts of overexpressed proteins are incubated with resin saturated with ligands. This is followed by filtration where bound protein stays on the resin, while unbound protein will pass through the filter. The amount of protein in the flow-through are analyzed by microfluidic capillary electrophoresis and is inversely correlated to the affinity of the interactions. In the proof-of-principle experiments, the authors benchmarked the method against 210 PDZ-peptide interactions of known affinities.

If aiming for the identification of interactions of potential biological relevance, it is crucial to confirm interactions in the context of the full-length proteins. Such validations can, for example, be made through the high-throughput luminescence-based mammalian interactome mapping (LUMIER) assays [77, 78], the mammalian protein-protein interaction trap (MAPPIT) [79], or yellow fluorescence protein-fragment complementation assay [80]. As reviewed recently, there is a growing number of approaches for studying and validating protein-protein interactions in cell signaling networks [81].

### Computational approaches

Complementing the experimental approaches, different computational approaches have been developed for the identification of motifs, such as SLiMFinder [82], DoReMi [83], and MotifHound [84]. To identify motifs in a given sequence, a combination of sequence properties is typically used such as i) a disorder propensity as motifs are enriched in disordered regions [85], ii) sequence conservation [86] and iii) a tendency to occur in functionally related proteins [82]. For example, a recent study on mitosis related proteins identified a new motif (Fx[ILV][FHY]x[DE]) termed the ABBA motif in the A type cyclins BUBR1, BUB1 and Acm1 [87].

While most approaches focus on the disorder property, Stein et al. took a structure based approach focusing on the fact that most motifs that are found in disordered regions will take defined structure(s) upon binding [88]. By scanning through the available protein complexes in the PDB, they discovered unnoticed peptide-based interactions and reported a list of novel peptide binding domains together with their recognition motifs. Following a structure- and data-based approach, De Bartolo and co-workers performed a genome-wide prediction of peptides binding to the human prosurvival Bcl-2 proteins. Predicted interactions were tested through SPOT arrays and in solution affinity measurements revealed affinities in the 1–500 nM K_D_ range [89].

Recently, Chen et al. performed a genome-wide prediction of motif-mediated interactions by taking advantage of the known motifs in the ELM database, analyzing structures of domain-motif complexes and using non-structural information such as the gene ontology similarities and phylogenetic profile similarities [90]. They provided a list of 79,000 new predicted domain-motif interactions, although without experimental validation. In the future, it will be interesting to follow how computational analysis and experiments together map out motifs in various proteomes.

## Conclusions

There is a plethora of experimental methods for the identification and characterization of domain-motif interactions (Table 2). Each method has its pros and cons, but together they provide complementary data. From our literature review it is clear that most of these methods have been developed for, and applied to, a limit set of ubiquitous domain families such as PDZ, WW, SH2 and SH3 domains, leaving many of the peptide binding domain families largely uncharted.

Interactions that rely on PTMs such as phosphorylation or acetylation are a challenge for most methods and there is a need for method development to allow for efficient identification of such interactions. Other challenges relate to fact that scaffold proteins often are composed of arrays of domains. Although information on the binding specificities of individual domains may be available, it does not necessary reflect the specificity of the domains in the context of the full-length proteins. In addition, connected domains of a bait protein might bind to linked motifs in a target protein, which may increase the apparent affinity and enhance the specificity of the interactions [91, 92]. Thus, dedicated approaches should be developed to account for such scenarios.

Nevertheless, by taking advantage of methods such as high-density peptide microarrays and proteomic display methods, and focusing the efforts on less explored peptide binding domain families it should be feasible to largely expand the knowledge on the binding motifs in the proteomes within the next ten years. By combining the finding from such efforts with the results of high-throughput Y2H and AP-MS we will obtain detailed maps of protein-protein interaction networks with assigned binding sites.

## Abbreviations

AP-MS: Affinity-purification coupled to mass spectrometry
ELISA: Enzyme-linked immunosorbent assay
ELM: Eukaryotic linear motif
ITC: Isothermal titration calorimetry
PDZ: Postsynaptic density protein 95/discs large/zona occludens 1
ProP-PD: Proteomic peptide phage display
PTB: Phosphotyrosine-binding
PTM: Post-translational modification
SH2: Src Homology 2
SH3: Src Homology 3
Y2H: Yeast-two-hybrid
